# Supplementary material for: Genomic and chemical insights into a human lectin-binding extracellular polysaccharides from Parageobacillus toebii strain H-70
Source: PLoS One. 2026 Jan 8;21(1):e0340423. doi: 10.1371/journal.pone.0340423 (PMC12782446; doi:10.1371/journal.pone.0340423)
Supplement: S1 File — (DOCX) [file pone.0340423.s001.docx]

***Genomic and chemical insights into a human lectin-binding*** ***extracellular polysaccharides from Parageobacillus toebii strain H­-70***

Diana Ghevondyan^1,2^, Armine Margaryan^1,2^, Fabrizio Chiodo^3,4^, Yvette van Kooyk^4^, Ilaria Finore^3*^, Annarita Poli^3^, Hovik Panosyan^2,5*^

^1^Department of Biochemistry, Microbiology and Biotechnology, Yerevan State University, Yerevan, Armenia

^2^Research Institute of Biology, Biology Faculty, Yerevan State University, Yerevan, Armenia;

^3^Institute of Biomolecular Chemistry, National Council of Research (C.N.R.), Pozzuoli, Naples, Italy

^4^Department of Molecular Cell Biology and Immunology, Amsterdam UMC, Vrije Universiteit Amsterdam, Amsterdam, The Netherlands

^5^Department of Biomedical Sciences, Institute of Pharmacy, Yerevan State University, Yerevan, Armenia

**^*^Corresponding authors**.

E-mail: [hpanosyan@ysu.am](mailto:hpanosyan@ysu.am) (HP)

E-mail: [ilaria.finore@cnr.it](mailto:ilaria.finore@cnr.it) (IF)

**S1 Table. dDDH values of *P. toebii* H-70 strain and other strains belong to *Parageobacillus*, *Geobacillus*, *Aeribacillus*, *Saccharococcus* and *Anoxybacillus* genera.**

| **Genome** | **dDDH**  **(%)** | **G+C difference (%)** |
| --- | --- | --- |
| *Parageobacillus toebii* DSM 14590^T^ | 92.3 | 0.53 |
| *Parageobacillus galactosidasius* DSM 18751^T^ | 91.4 | 1.31 |
| *Parageobacillus yumthangensis* [MTCC](https://www.ncbi.nlm.nih.gov/biocollections?term=MTCC%5BUnique%20institution%20code%5D) 12749^T^ | 84.6 | 0.55 |
| *Aeribacillus* *pallidus* [KCTC 3564](https://kctc.kribb.re.kr/en/collection/view?sn=3564)^T^ | 45.2 | 3.57 |
| *Saccharococcus thermophilus* DSM 4749^T^ | 36.2 | 1.99 |
| *Parageobacillus thermantarcticus* DSM 9572^T^ | 35.6 | 0.78 |
| *Parageobacillus caldoxylosilyticus* DSM 12041 ^T^ | 35.3 | 1.06 |
| *Parageobacillus thermoglucosidasius* DSM 2542 ^T^ | 34.6 | 1.0 |
| *Anoxybacillus karvacharensis* DSM 106524^T^ | 30.2 | 1.31 |
| *Anoxybacillus flavithermus*[DSM 2641](https://www.dsmz.de/collection/catalogue/details/culture/DSM-2641)^T^ | 26.5 | 1.11 |
| *Geobacillus thermodenitrificans* DSM 465^T^ | 25.9 | 6.16 |
| *Anoxybacillus tepidamans* DSM 16325^T^ | 24.9 | 0.37 |
| *Geobacillus* *subterraneus* [KCTC 3922](https://kctc.kribb.re.kr/en/collection/view?sn=3922)^T^ | 23.9 | 9.31 |

**S2 Table. Genetic determinants of EPS biosynthesis pathways in *P. toebii* strain H-70.**

| **Peg. numbers** | **Gene product** |
| --- | --- |
| ***Transport of sugars*** | |
| Peg.1935; 1936 | ABC transporter, substrate-binding protein (cluster 2, ribose/xylose/arabinose/galactose) |
| Peg.1937 | ABC transporter, permease protein (cluster 2, ribose/xylose/arabinose/galactose) |
| Peg.1463; 1465; 3044 | ABC transporter, permease protein 1 (cluster 1, maltose/g3p/polyamine/iron) |
| Peg.1464; 3043 | ABC transporter, ATP-binding protein (cluster 1, maltose/g3p/polyamine/iron) |
| Peg.167 | Putative sodium-glucose/galactose cotransporter |
| Peg.389; 827 | PTS system, glucose-specific IIA component (EC 2.7.1.199) |
| Peg.44 | PTS system, fructose-specific IIA component (EC 2.7.1.202) / PTS system, fructose-specific IIB component (EC 2.7.1.202) / PTS system, fructose-specific IIC component |
| Peg.1806 | PTS system, N-acetylglucosamine-specific IIC component / PTS system, N-acetylglucosamine-specific IIB component (EC 2.7.1.193) |
| ***Sugar utilization*** | |
| Peg.1657 | Fructokinase (EC 2.7.1.4) |
| Peg.436 | Fructose-1,6-bisphosphatase, GlpX type (EC 3.1.3.11) |
| Peg.369 | Mannose-6-phosphate isomerase (EC 5.3.1.8) |
| Peg.43 | 1-phosphofructokinase (EC 2.7.1.56) |
| Peg.2748 | Glutamine-fructose-6-phosphate aminotransferase [isomerizing] (EC 2.6.1.16) |
| Peg.1804 | Glucosamine-6-phosphate deaminase (EC 3.5.99.6) |
| Peg.1959 | Galactose-1-phosphate uridylyltransferase (EC 2.7.7.10) |
| Peg.1248 | Glucokinase (EC 2.7.1.2) |
| Peg.827 | Glucose-1-phosphate adenylyltransferase (EC 2.7.7.27) |
| Peg.1957 | Galactokinase (EC 2.7.1.6) |
| Peg.1742 | Glucose-6-phosphate 1-dehydrogenase (EC 1.1.1.49) |
| Peg.3109 | Glucose-6-phosphate isomerase (EC 5.3.1.9) |
| Peg.1944; 433 | Fructose-bisphosphate aldolase class II (EC 4.1.2.13) |
| Peg.3648 | Glutamine-fructose-6-phosphate transaminase (isomerizing), isomerase subunit (EC 2.6.1.16) |
| Peg.2321 | Galactose 1-dehydrogenase (EC 1.1.1.48) |
| Peg.2317; 1589 | Phosphoglucomutase (EC 5.4.2.2) Phosphomannomutase (EC 5.4.2.8) |
| Peg.2747 | Phosphoglucosamine mutase (EC 5.4.2.10) |
| Peg.2320 | Mannose-1-phosphate guanylyltransferase / Phosphomannomutase (EC 5.4.2.8) |
| ***Synthesis of Sugar Nucleotide Precursors*** | |
| Peg.79 | UDP-N-acetylglucosamine 4,6-dehydratase (inverting) (EC 4.2.1.115) |
| Peg.3476 | UDP-N-acetylglucosamine 4,6-dehydratase (EC 4.2.1.135) |
| Peg.1681 | N-acetylmannosaminyltransferase (EC 2.4.1.187) |
| Peg.2860 | N-acetylglucosamine-1-phosphate uridyltransferase (EC 2.7.7.23) / Glucosamine-1-phosphate N-acetyltransferase (EC 2.3.1.157) |
| Peg.1677 | UDP-N-acetylglucosamine 2-epimerase (EC 5.1.3.14) |
| Peg.1680 | UDP-N-acetyl-D-mannosamine dehydrogenase (EC 1.1.1.336) |
| Peg.1758 | ADP-ribose pyrophosphatase (EC 3.6.1.13) |
| Peg.3430; 1579 | UTP-glucose-1-phosphate uridylyltransferase (EC 2.7.7.9) |
| Peg.1628 | UDP-glucose 6-dehydrogenase (EC 1.1.1.22) |
| Peg.1958; 1590 | UDP-glucose 4-epimerase (EC 5.1.3.2) |
| Peg.3441 | Undecaprenyl-phosphate alpha-N-acetylglucosaminyl 1-phosphate transferase (EC 2.7.8.33) |
| Peg.1580 | Undecaprenyl-phosphate galactosephosphotransferase (EC 2.7.8.6) |
| ***EPS biosynthesis and secretion*** | |
| Peg.1572 | Tyrosine-protein kinase transmembrane modulator EpsC |
| Peg.1573 | Tyrosine-protein kinase EpsD (EC 2.7.10.2) |
| Peg.1576 | Manganese-dependent protein-tyrosine phosphatase (EC 3.1.3.48) |
| Peg.3439 | Glycosyltransferase (EC 2.4.1.-) |
| Peg.1900 | UDP-N-acetylglucosamine:L-malate glycosyltransferase |
| Peg.1503 | oligosaccharide flippase (Wzx) family protein |
| Peg.1601 | oligosaccharide repeat polymerase |
| Peg.1128 | TPR repeat-containing protein YrrB |
| Peg.191; 1244 | Putative membrane peptidase, contains TPR repeat domain |
| Peg.1887 | FIG009300: TPR-repeat-containing protein |
| Peg.3533 | TPR repeat-containing protein YvcD |
| Peg.61 | TPR domain protein, putative component of TonB system |
| ***Polymer modification*** | |
| Peg.280; 1402; 2227; 2418; 2949; 3090; 3104; 3244; 3251; 3293 | Acetyltransferase, GNAT family |
| Peg.297 | Acetyltransferase |
| Peg.404 | Phosphate acetyltransferase (EC 2.3.1.8) |
| Peg.2567; 2979; 3093; 3409; 3089 | Uncharacterized N-acetyltransferase YkwB; YqjY; YkkB |
| Peg.3523 | Putative acetyltransferase YvoF |
| Peg.1530; Peg.1531 | Polysaccharide deacetylase, possible chitooligosaccharide deacetylase (EC 3.5.1.41) |
| Peg.3438 | Polysaccharide pyruvyl transferase CsaB |
| Peg.3606 | Probable polysaccharide deacetylase pdaB precursor |
| ***Regulator system*** | |
| Peg.1805 | Predicted transcriptional regulator of N-Acetylglucosamine utilization, GntR family |
| Peg.42 | Transcriptional repressor of the fructose operon, DeoR family |
| Peg.2141; 3045 | Maltose operon transcriptional repressor MalR, LacI family |
| Peg.652 | Transcriptional regulator in cluster with unspecified monosaccharide ABC transport system |
| Peg.5; 1255; 3608 | Transcriptional regulator |
| Peg.41; 1375;2311; 2412; 2772 | Two-component transcriptional response regulator, LuxR family |
| Peg.312; 391; 988; 1510; 2364; 2750 | Transcriptional regulator, MerR family |
| Peg.426; 1398; 1515; 1661; 2415; 2554; 3036 | Transcriptional regulator, AcrR family |
| Peg.821 | Response regulator of the LytR/AlgR family |
| Peg.875 | Transcriptional regulator, DeoR family |
| Peg.601; 1736 | Positive regulator of CheA protein activity (CheW) |
|  |  |
| Peg.1272 | Xylose-responsive transcription regulator, ROK family |
| Peg.1343; 2249; 3247 | Transcriptional regulator, LacI family |
| Peg.1353 | Transcriptional regulator, RpiR family |
| Peg.1622; 2013 | Transcriptional regulator, ArsR family |
| Peg.1672; 2739 | Transcriptional regulator, Xre family |
| Peg.1971 | Transcriptional regulator, LysR family |
| Peg.2092 | Uncharacterized transcriptional regulator YhgD, TetR family |
| Peg.751; 1653; 2135; 3226; 3255 | Transcriptional regulator, AraC family |
| Peg.2423; 3292 | Transcriptional regulator, MarR family |
| Peg.2758 | Transcriptional regulator, PadR family |
| Peg.2815; 2816 | Two-component system YycFG regulatory protein YycH; YycI |
| Peg.2873 | Transition state regulatory protein AbrB |
| Peg.2947 | Regulatory protein RecX |
| Peg.3446 | Two-component transcriptional response regulator DegU, LuxR family |
| Peg.254;257; 1613; 1652; 1926; 1927; 2411 | Two-component system sensor histidine kinase |
| Peg.375 | Cell envelope stress response system LiaFSR, sensor histidine kinase LiaS(VraS) |
| Peg.600 | Signal transduction histidine kinase CheA |
| Peg.1206 | Transcriptional repressor CcpN, MarR family |
| Peg.1752; 2279 | Transcriptional repressor CzrA, ArsR family |

**
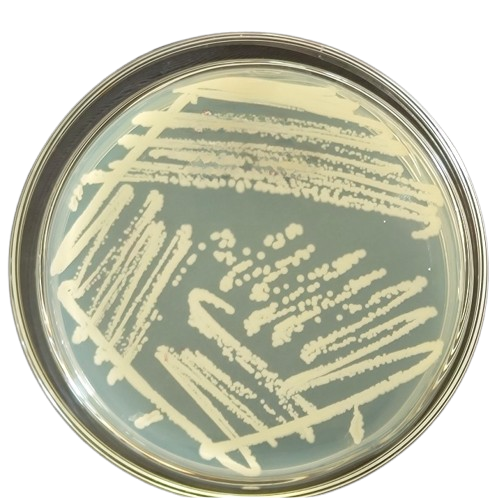
**

**S1 Fig. The colonies of strain H-70 on minimal medium A enriched by sucrose 2% (w/v) after 48 h cultivation. Mucous consistency of the strain due to EPS production.**

**
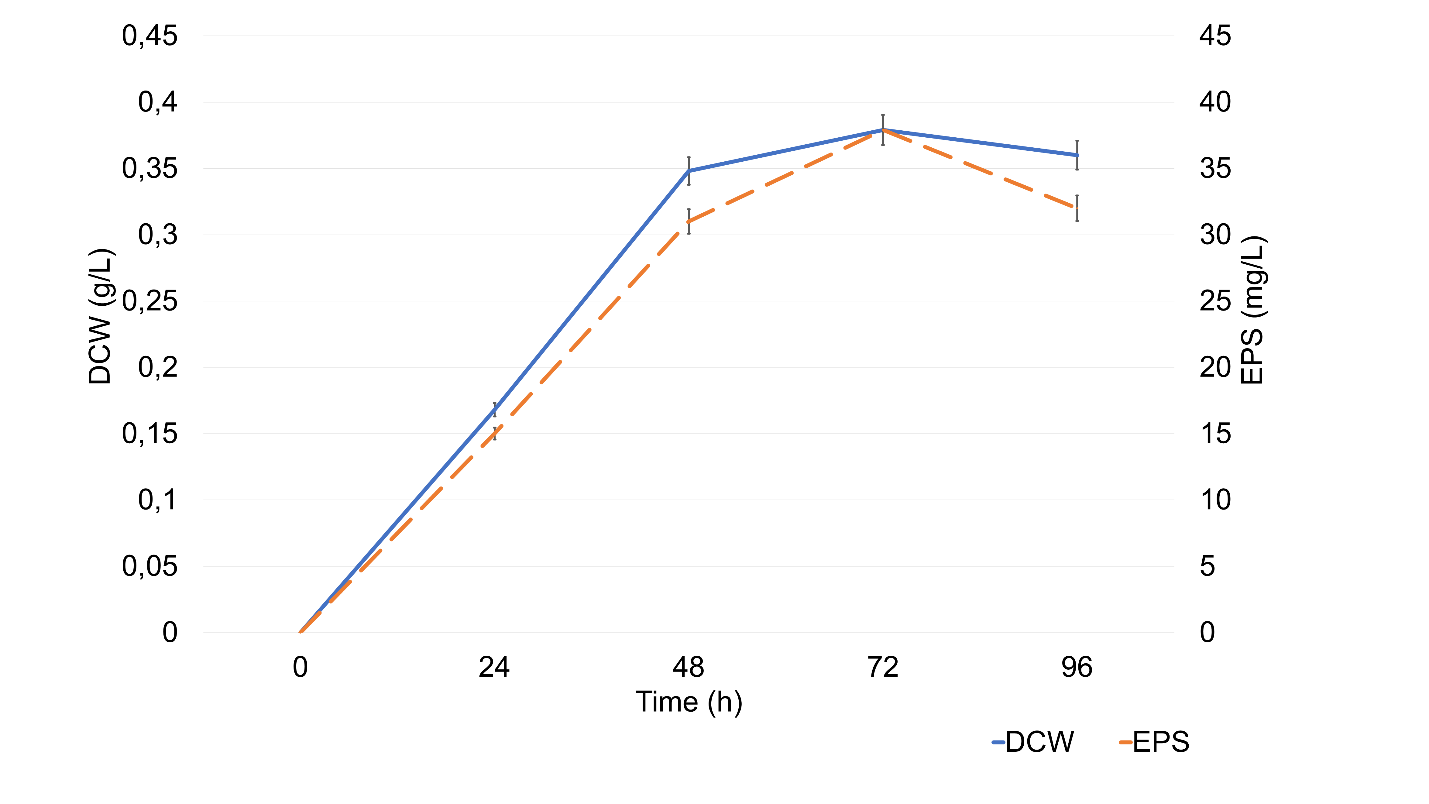
**

**S2 Fig. Time course of growth and EPS production by strain H-70 in sucrose-supplemented minimal medium A with at 55 °C, pH 7.0. Samples were taken in 6 h intervals and assayed for growth (blue line) and EPS content (red line)**

**
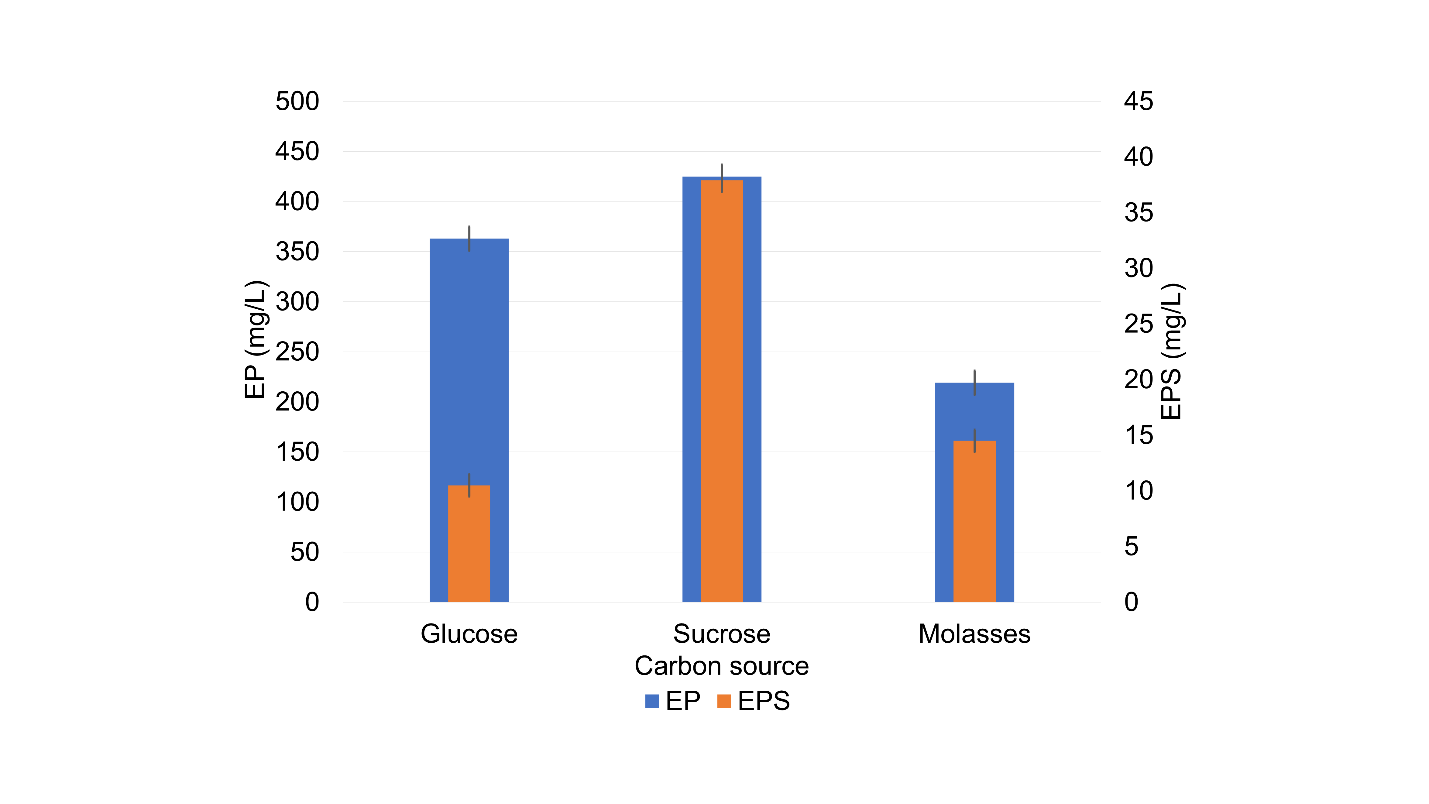
**

**S3 Fig. EP and EPS production by the strain H-70 from glucose, sucrose and molasses at the stationary phase of growth.**

*
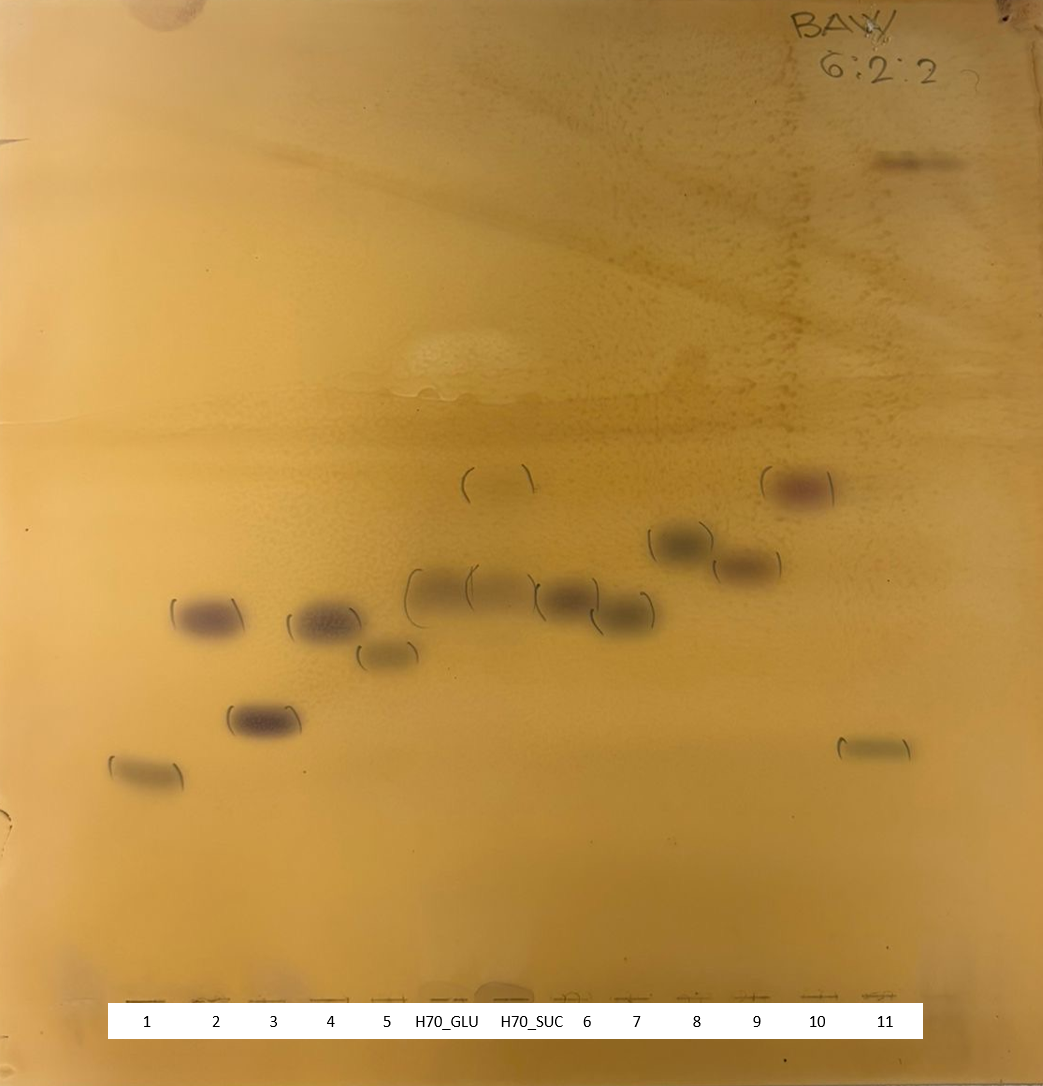
*

**S4 Fig. TLC analyses of hydrolysate EPS from strain H-70 in comparison with monomer standards.** 1- Galacturonic acid; 2- Fructose; 3- Trehalose; 4- Glucose; 5- Galactose; H-70-GLU- Hydrolysate acid of EPS from strain H-70 grown on glucose; H-70-SUC- Hydrolysate acid of EPS from strain H-70 grown on sucrose; 6- Mannose; 7-Arabinose; 8-Xylose; 9- Fucose; 10- Rhamnose; 11- Glucuronic acid. (TLC solvent systems: *n*-BuOH/AcOH/H_2_O 6:2:2, by vol.).


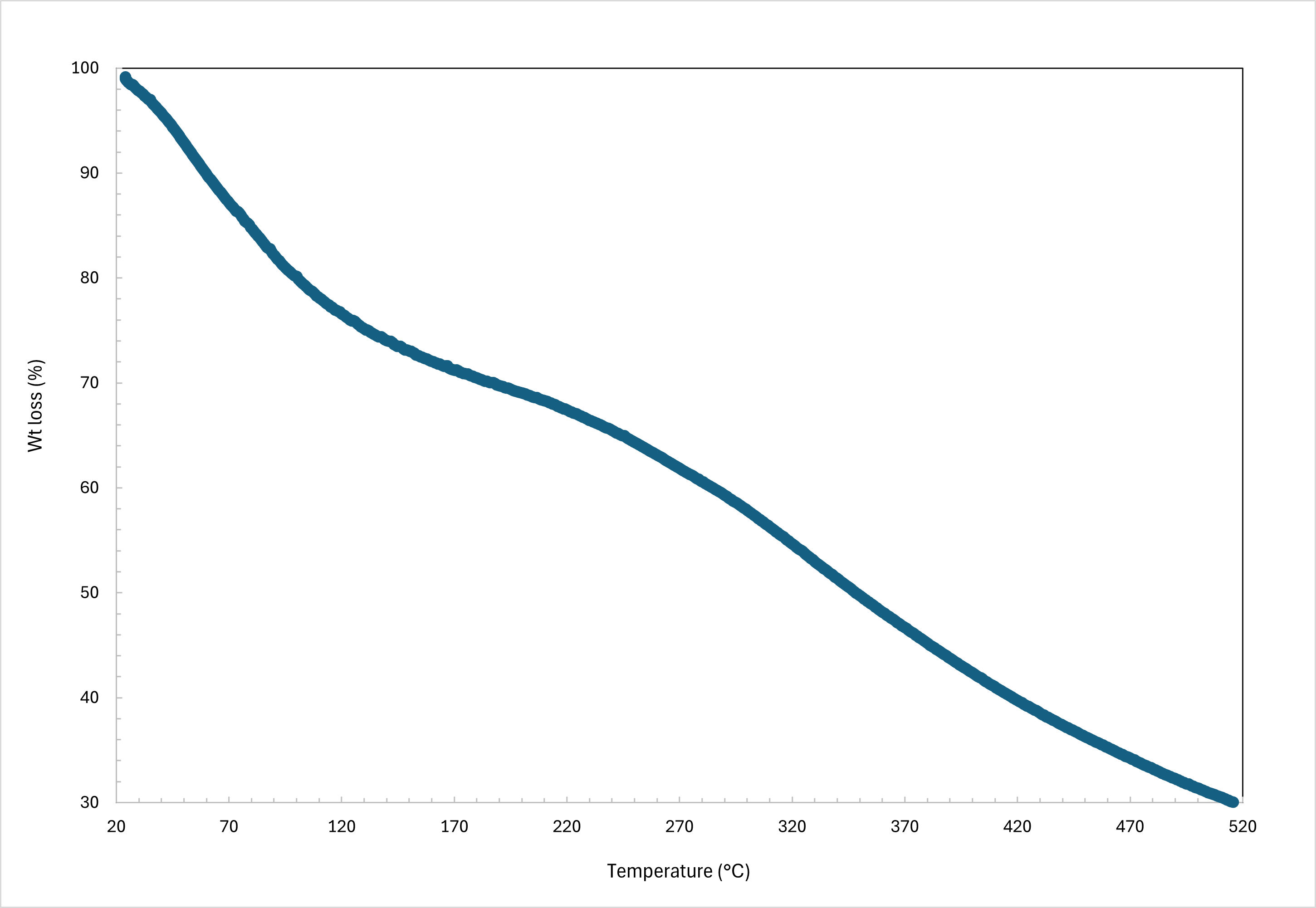


**S5 Fig. Thermogravimetrical analysis of EPS from strain H-70 grown in sucrose containing medium.**


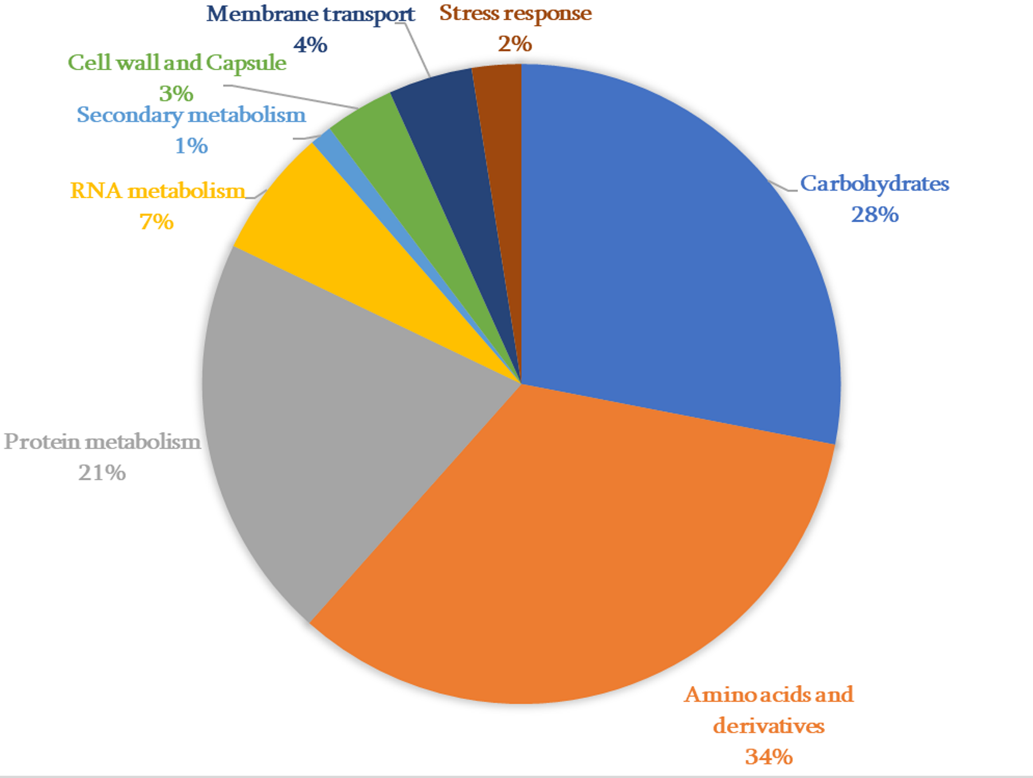


**S6 Fig. Categorization of CDSs into subsystems based on RAST annotation.**


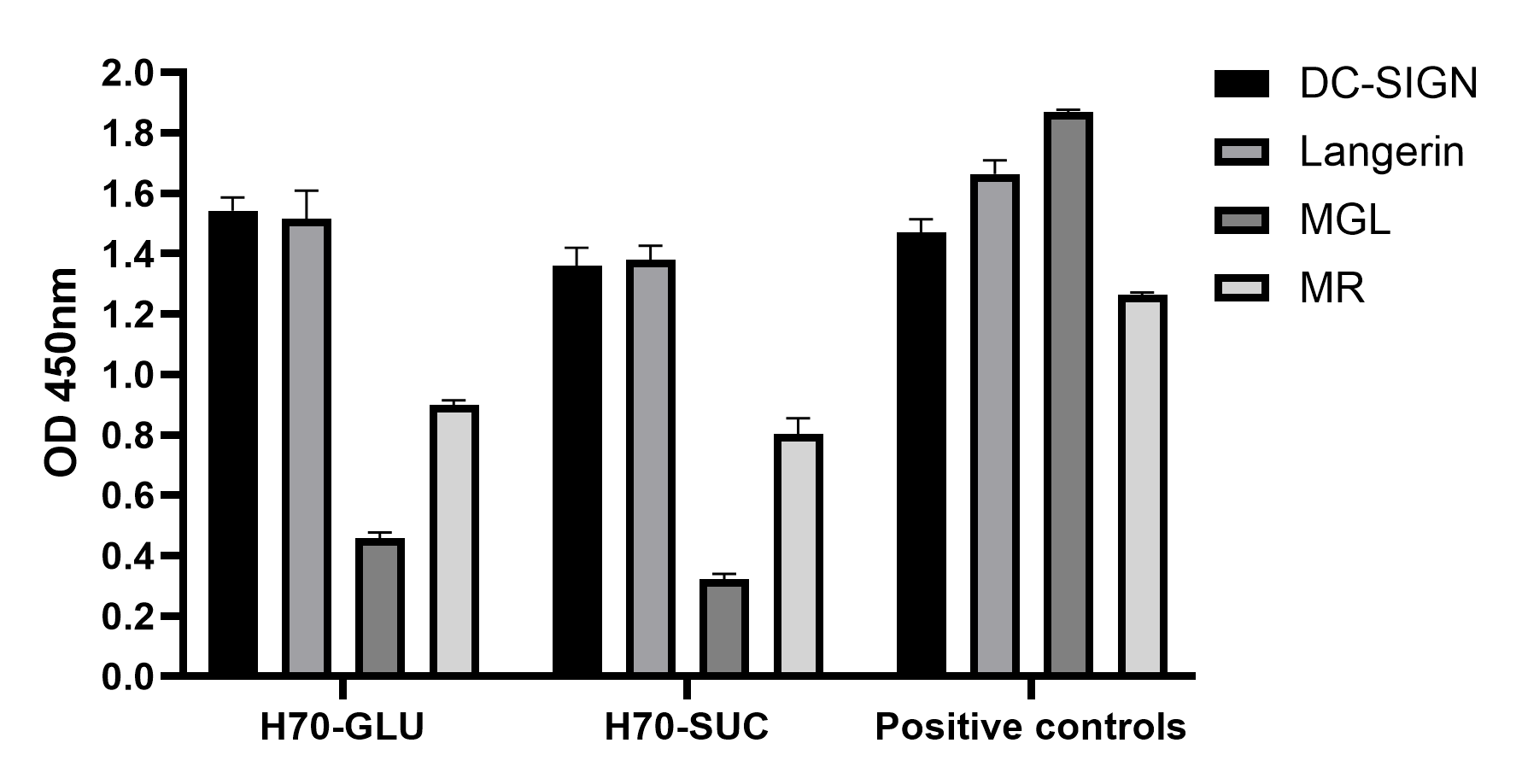


**S7 Fig. Binding of EPS produced in glucose- or sucrose-enriched media was assessed for DC-SIGN, Langerin, MGL, and MR using a solid-phase assay in calcium-containing buffer**. Data points represent the mean of duplicate measurements measured as optical density (OD) at λ 450nm. Error bars represent standard deviation.
